# Can back exosuits simultaneously increase lifting endurance and reduce musculoskeletal disorder risk?

**DOI:** 10.1017/wtc.2024.8

**Published:** 2024-11-28

**Authors:** K.M. Rodzak, P.R. Slaughter, D.N. Wolf, C.C. Ice, S.J. Fine, K.E. Zelik

**Affiliations:** 1 Department of Mechanical Engineering, Vanderbilt University, Nashville, TN, USA; 2 Department of Biomedical Engineering, Vanderbilt University, Nashville, TN, USA; 3 Department of Physical Medicine & Rehabilitation, Vanderbilt University, Nashville, TN, USA

**Keywords:** exoskeletons, lifting, low back disorder, military, performance augmentation

## Abstract

The objectives of this case series study were to test whether an elastic back exosuit could increase a wearer’s endurance when lifting heavy objects and to assess whether lifting more cancels out the exosuit’s risk reduction benefits. We found that 88% of participants increased their lifting repetitions while wearing an exosuit, with endurance increases ranging from 28 to 75%. We then used these empirical data with an ergonomic assessment model based on fatigue failure principles to estimate the effects on cumulative back damage (an indicator of low back disorder risk) when an exosuit is worn and more lifts are performed. Participants exhibited 27–93% lower cumulative back damage when wearing an exosuit. These results confirmed that wearing an exosuit increased participants’ lifting capacity without canceling out injury risk reduction benefits. Back exosuits may make it possible to simultaneously boost productivity and reduce musculoskeletal disorder risks, which is relevant to workers in civilian and defense sectors.

## 1. Introduction

Back pain and injuries are the most common types of work-related musculoskeletal disorders (Bureau of Labor Statistics 2020). These often occur within fatiguing jobs that require repetitive or heavy lifting due to high loading and overexertion of the back.

Back exos are emerging wearable technologies designed to reduce back pain, injuries, and fatigue. The term exo refers to a wearable device that augments, assists, or enhances human movement or posture and encompasses both rigid exoskeletons and soft exosuits, and both powered (active/motorized) and elastic (passive) devices. Exos complement other ergonomic interventions and can be used in environments where automation or traditional ergonomic controls are impractical. Back exos provide an assistive moment about the lumbar spine and hips when a user lifts. This exo moment, also commonly referred to as the exo assistance, can reduce back strain, fatigue, spinal compression forces, and musculoskeletal disorder risk factors based on various modeling, laboratory, and field studies (e.g., Kermavnar et al. 2021; Lamers and Zelik 2021; Zelik et al. 2022; dos Anjos et al. 2022).

Back exos may also increase a user’s lifting endurance; however, existing evidence is mixed and limited. Lifting endurance is one indicator of physical capacity to do work and is defined as the number of lifts a user can perform at a given rate or within a given time. Two prior studies found increases in lifting endurance of 11–30% when wearing a back exo (Tan et al. 2019; Baltrusch et al. 2020), while two other studies found no statistical difference in lifting endurance with versus without a back exo (Kozinc et al. 2021; So et al. 2022). These four studies involved different exos (e.g., powered versus elastic devices), lifting postures (e.g., squat, stoop, and freestyle), and lifted weights (5–20 kg), so it is difficult to generalize these findings or even compare the quantitative results between studies.

There is a need for more research on back exo effects on lifting endurance to expand the evidence base and address current knowledge gaps. For instance, no exo studies have been conducted on lifting endurance when handling heavy objects (e.g., over 20 kg). Understanding the effect of back exos on heavy lifting is important for various industries where these duties are commonplace, such as in logistics, distribution, and baggage handling. A quintessential example we encountered was with U.S. Army Soldiers in field artillery and distribution units. These individuals often lift 20–60 kg objects, and these groups exhibit high rates of back overuse injury (Reynolds et al. 2002; Hollander and Bell 2010; Gun et al. 2022). Across the Army, an average of 460 Soldiers are diagnosed with a back overuse injury every day (U.S. Army Public Health Center 2016, 2017, 2018, 2019). However, these Soldiers also need to sustain a high level of physical performance (e.g., during multiday missions). Soldiers and civilians who perform strenuous lifting, loading, and unloading jobs could benefit from wearable technologies that both reduce their musculoskeletal injury risks and increase their physical endurance (Mudie et al. 2018; Fox et al. 2019; Golabchi et al. 2022). While increasing physical endurance is desirable, there is a knowledge gap related to whether lifting more might cancel out the injury risk reduction benefits of a back exo.

In this study, we sought to address these knowledge gaps related to exo effects on lifting endurance and injury risk. One objective was to test empirically whether a back exo – specifically, an elastic exosuit – can increase endurance when lifting heavy objects. A second objective was to apply ergonomic modeling to assess whether performing more lifting repetitions cancels out the risk reduction benefits of an exo. Thus, the first objective evaluated the feasibility of heavy lifting augmentation and provided empirical data on the range of potential exo effects, while the second sought to gain generalizable insights on the interrelationship between exo assistance, lifting repetitions, and low back disorder risk during material handling. Throughout this article, we use the term exo when describing prior research or study results that apply broadly to this class of back-assist device, and we use the term exosuit when referring to results or characteristics that are specific to the type of device tested empirically in this study.

## 2. Methods

### 2.1. Participants

A total of nine individuals volunteered and were consented to participate in this study (participant demographics provided in subsequent sections). These participants were from field artillery units within the 101st Airborne Division of the U.S. Army. Field artillery Soldiers were tested in this study because we previously identified them as a group within the Army at high risk of back overuse injuries, and because they were involved in the development of the SABER exosuit we tested (Slaughter et al. 2023). The protocol was approved by the Vanderbilt University Institutional Review Board and the U.S. Army Human Research Protections Office. Commanding officers were not involved in or present during study recruitment, consent, or introduction to avoid undue influence.

### 2.2. Experiment overview

Participants performed repeated lifting until failure, with versus without a passive elastic back exosuit, to assess the effects on lifting endurance. Two case series studies were performed on separate days. Originally, we planned for these case series to be identical, but key learnings from the first case series resulted in us altering and expanding the protocol in the second to improve the rigor and interpretability of the results. Specific methods are detailed in each case series below. Each case series data collection was completed on a single day to avoid between-day variability and dropouts.

**Figure 1.**
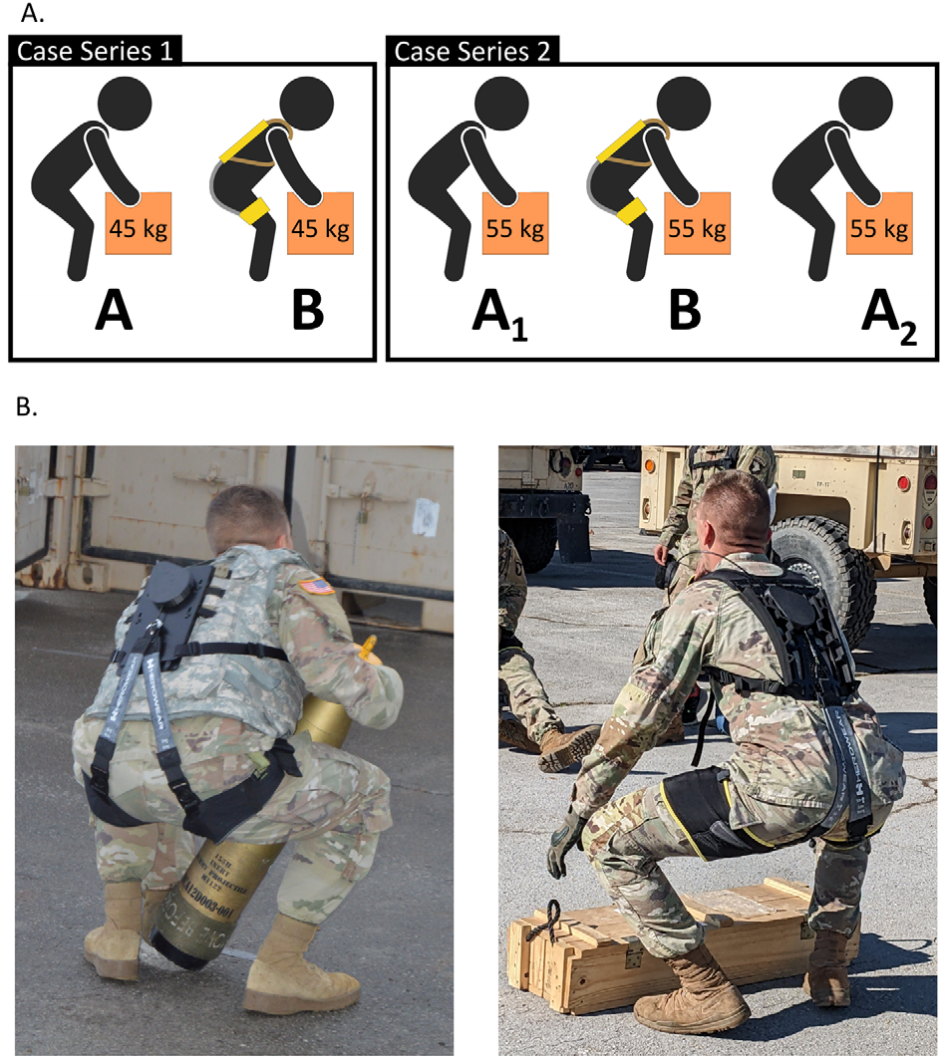
(A) Overview of lifting endurance tests. In case series 1, participants performed an AB test in which they lifted 45 kg repeatedly until failure without the exosuit and then performed this task while wearing the exosuit. In case series 2, participants performed an ABA test in which they lifted 55 kgs repeatedly until failure without the exosuit, then with the exosuit, and then again without the exosuit. (B) The photos show representative participants wearing the exosuit during case series 1 (left) and 2 (right), as well as the operationally relevant objects they lifted.

During the exosuit condition, the participants wore a SABER prototype (Figure 1). SABER is an unpowered back exosuit that contains no motors or batteries (Slaughter et al. 2023). This exosuit is comprised of a harness (upper-body interface), thigh sleeves (lower-body interface), clutch-switch system (to toggle assistance on and off), and elastic bands (along the back that act as an artificial set of back and hip muscles). SABER uses elastic bands to biomechanically assist when users bend forward or lift (Lamers et al. 2018, 2020). We fit each participant with a SABER prototype and trained them on how to use the device prior to testing. Participants were then given 10–20 min to wear the exosuit and perform practice bends and lifts to acclimate.

SABER is functionally similar to the HeroWear Apex (HeroWear 2020; Goršič et al. 2021; Kang and Mirka 2023) but was completely redesigned to integrate with a Soldier’s standard gear. The SABER exosuit was designed as part of the Army Pathfinder program. See Slaughter et al. (2023) for a detailed discussion of the SABER exosuit design, Soldier requirements, Pathfinder program, and where exos may fit within military technology and operations.

### 2.3. Case series 1

Five participants (all male, age 24.5 ± 6.1 years, height 1.8 ± 0.06 m) consented for case series 1, but one was excluded for reasons detailed below. We used an AB study design (Figure 1A) to test endurance first without the exosuit (A) versus second with the exosuit (B). The task was to lift a 155-mm field artillery round (45 kg) every 6 s until failure (i.e., until they could no longer lift at the prescribed rate). Participants completed both the control lifting set (A) and exosuit lifting set (B) in their standard issue uniforms and wore Improved Outer Tactical Vests (IOTVs). Participants lifted the artillery round the way they would during ammunition loading and unloading by squatting down, placing one hand at the base of the round and placing the other hand near the top end of the round, as can be seen in Figure 1B. We instructed the participants to lift the round every 6 s (10 lifts/min) in accordance with a metronome. This pace was chosen based on pilot testing to sufficiently exhaust participants over several minutes. This frequency has also been used in other lifting studies (Genaidy and Asfour 1989; Potvin and Norman 1993). Lifts were counted by a member of the research team, and we confirmed these counts via video taken during testing. The set was over when the participant said they were done or unable to continue lifting at the prescribed pace. Participants were then given 30 minutes of rest. This rest duration was chosen based on participant availability and pilot testing, and was similar to the rest time used in other lifting studies (Bensel et al. 2008; So et al. 2022). At the end of the rest period, participants donned the exosuit. They then completed the next lifting set with the exosuit in engaged (assistance) mode. During testing, participants were put in groups of 2–3 and started the sets synchronously together, with the initial thought that this group workout approach might be more encouraging and motivating to participants. Unfortunately, motivation is bidirectional, and we observed one participant give up early (for reasons unrelated to fatigue) when another participant in the group reached his fatigue limit and stopped. This former participant’s data were therefore excluded from our analysis. For each other participant (*N* = 4), we computed the percentage change in the number of lifts performed with versus without the exosuit.

### 2.4. Case series 2

Four participants (all male, age 21.8 ± 3.6 years, height 1.8 ± 0.04 m) were consented in case series 2. We used an ABA withdrawal study design (Figure 1A) to test endurance first without the exosuit (A_1_, first control) versus second with the exosuit (B, intervention) versus third without the exosuit (A_2_, second control). The second control set was added to this case series to assess if lifting endurance returned back to baseline levels after the exosuit was removed. If so, this would provide more compelling evidence that the exosuit was the cause of endurance changes in condition B, as opposed to other factors (e.g., learning, random chance, and fatigue). The task was to lift a box of two 105-mm rounds (55 kg total box weight) every 6 s until failure. Participants wore their standard issue uniforms but did not wear IOTVs. Participants lifted the box the way they would during ammunition loading and unloading by squatting down and grabbing the handles on the ends of the box, as can be seen in Figure 1B. Participants were instructed to lift the box every 6 s in accordance with a metronome. All lifting was recorded, and lifts were counted based on the video after testing. The set of lifts ended when the participant said they were done or unable to continue lifting at the prescribed pace. After each set, they were given 20 min to rest. At the end of the first rest period, participants donned the exosuit and completed the next lifting set with exosuit assistance engaged. Afterward, they removed the exosuit and were given 20 min to rest. They then completed a third set of lifting without the exosuit. For this case series, we staggered the lifting start times so that no two individuals began sets at the same time. We made this adjustment to the protocol after we observed in case series 1 that one participant chose to stop when a group member reached his limit and stopped. For each participant in case series 2 (*N* = 4), we computed the percentage change in the number of lifts performed with versus without the exosuit (i.e., B relative to A_1_, B relative to A_2_, and B relative to the average of A_1_ and A_2_).

### 2.5. Injury risk modeling

We used data from case series 1 and 2 with a previously established ergonomic assessment tool to model the effects on low back disorder risk when the exosuit is worn and more lifts are performed. The ergonomics assessment tool used was Exo-LiFFT, which is based on mechanical fatigue failure principles that are believed to underlie overexertion injuries (Gallagher et al. 2017; Zelik et al. 2022). We selected Exo-LiFFT because it was previously developed to evaluate the effect of back exos on injury risk, whereas it currently remains less clear how to adapt other ergonomic assessment tools (e.g., Revised NIOSH Lifting Equation, Snook Tables) to be compatible with exos. Below we summarize how we used Exo-LiFFT and we refer readers to Zelik et al. (2022) for an extended discussion of this and other potential risk assessment tools for back exos. Empirical data collected during the case series and secondary experiments (detailed below and in Appendix B) were used to generate inputs to Exo-LiFFT. We then performed two modeling evaluations. The first estimated the exosuit’s effects on injury risk based on the empirical data collected in the case series. The second was a parameter sweep to explore the interaction more broadly between exo assistance and lifting repetitions, and their effects on injury risk.

### 2.6. Modeling evaluation 1

We used empirical case series data to assess whether performing more lifting repetitions with the exosuit canceled out the risk reduction benefits of the exosuit. For each participant in each case series, we computed the cumulative damage (an indicator of low back disorder risk) for the A lifting set (without exosuit), for the B lifting set (with exosuit), and the percentage change in cumulative damage with versus without the exosuit.

The ergonomic assessment tool Exo-LiFFT uses the object weight, lifting repetitions, peak load moment, and exo moment to estimate indicators of musculoskeletal injury risk (e.g., low back disorder risk and cumulative damage). Object weight was known for each case series. Lifting repetitions were measured in each case series, for exosuit and no exosuit sets, respectively. Peak load moment serves as a practical surrogate metric for peak loading on the low back during lifting. It is computed as the object’s weight multiplied by the maximum horizontal distance from the object to the hip (or lumbar spine), which generally occurs at the deepest part of the lift. Before the first lifting set, participants were instructed to get into position as if they were about to lift the object, but then to hold their position (i.e., at the deepest part). We used a tape measure to find the horizontal distance from the object to the hip. Next, we estimated exo moment. From this same body position, we measured the stretch of the exosuit’s elastic bands, which also reach their maximum stretch (and force) at the deepest part of the lift.

Elastic band stretch was used in combination with other data to compute the exo moment about the low back. These other data and methods are explained in full in Appendix B and are briefly summarized here. The maximum elastic band stretch, which is a function of trunk and hip flexion during lifting, was combined with force-displacement curves for the elastic bands (provided by the manufacturer of the bands) to compute the maximum force exerted by the exosuit. The exosuit’s moment arm about the lumbar spine was estimated using a database of digitized CT scans to find the distance from the center of the L5/S1 joint to the skin surface of the low back, then using physical measurements to find the distance from the skin to the bands. We multiplied the maximum elastic band force by the moment arm to calculate the peak lumbar extension moment generated by the exosuit. We then accounted for the lumbar flexion moment created by the trunk-worn weight of the exosuit. Finally, we applied data from secondary experiments that enabled us to account for elastic band hysteresis (energy loss) and slight differences in timing between peak exo moment and peak lumbar loading. Collectively, these enabled us to estimate the exo moment input to Exo-LiFFT. This input signifies how much the exo reduces peak loading on the low back (i.e., reduction in peak lumbar moment).

Exo-LiFFT outputs an estimate of expected cumulative damage to the low back based on a specified amount of loading. Cumulative damage refers to the initiation and growth of micro-cracks or micro-tears within musculoskeletal structures (e.g., bones, muscles, ligaments, and discs) due to mechanical fatigue (or creep) processes that are believed to underly overexertion injuries (Gallagher et al. 2017). In effect, cumulative damage is a way to quantify what we would colloquially call wear-and-tear. Cumulative damage is calculated by estimating the loading on a specific musculoskeletal structure or area of the body (i.e., the low back in this study), and then applying a previously established relationship between musculoskeletal loading and damage accumulation. This relationship is derived from empirical fatigue testing on cadaveric specimens (Gallagher et al. 2017; Zelik et al. 2022). For extended methodological details on Exo-LIFFT, see Appendix B. This is the main injury risk indicator we used to compare lifting with versus without the exosuit. We did not use the probability of being a high-risk job metric (termed low back disorder risk in Zelik et al. 2022), which is another output from Exo-LiFFT, as the primary outcome because some lifting sets in our study exceeded the validated bounds of this particular risk metric due to the heavy and bulky objects lifted by Soldiers (Gallagher et al. 2017). In contrast, cumulative damage is an indicator of wear-and-tear based on mechanical fatigue failure principles (Gallagher and Heberger 2015), which we expect to be applicable to a higher range of forces (Brinckmann et al. 1988) and for different musculoskeletal tissues (Carter and Caler 1985; Schechtman and Bader 1997). For each participant, we computed the change in cumulative damage with versus without the exosuit. We present these results alongside participant-specific increases in lifting endurance (repetitions) to address the secondary objective.

### 2.7. Modeling evaluation 2

We performed additional parameter sweeps to more broadly assess how exo moment and lifting repetitions interact to affect changes in cumulative damage. Specifically, we sought to better understand exo effects under other lifting conditions (e.g., lighter-weight, higher-repetition lifting) and with differing levels of back assistance from an exo. Although our empirical study (detailed above) focused on increasing physical performance by increasing lifting repetitions, another way to increase performance would be to perform the same number of repetitions but lift heavier objects. Therefore, we used parameter sweeps to better understand the implications of both increasing object weight and increasing lifting repetitions while wearing an exo.

We performed four complementary parameter sweeps. The first explored what happens when increasing the number of lifts from 0% (i.e., corresponding to a nominal number of lifts) to 100% (i.e., doubling the number of lifts performed). This range encompasses the lifting repetition increases observed in our case series testing. We then explored what happens when increasing the weight of the object lifted from 0% (i.e., a nominal weight) to 100% (i.e., doubling the weight). We performed these modeling sweeps using three different nominal weights (5, 23, and 45 kg). For all parameter sweeps, we varied the exo moment about the lumbar spine from 0 to 50 Nm, which encompasses the max assistance provided in case series 1 and 2 and encompasses most current commercial back exos (Di Natali et al. 2021; Madinei et al. 2022; Kang and Mirka 2023). For the model results presented, we used a nominal object-to-hip distance of 60 cm. We also confirmed that for other object-to-hip distance values (within a reasonable range for lifting, based on human anthropometrics) the general model trends and conclusions remain the same.

### 2.8. Case series analysis and summary metrics

For each case series, we counted the number of participants who increased versus decreased their lifting repetitions and who increased versus decreased cumulative damage while wearing the exosuit. As supplementary summary metrics, we computed the range and mean of these exosuit effects. For case series 2, we also quantified the reversal effects. Specifically, we counted the number of participants whose lifting repetitions (or cumulative damage) increased (or decreased) when the exosuit was donned (B relative to A_1_) and then decreased (or increased) when the exosuit was doffed (A_2_ relative to B). Confirming this reversal effect provides more confidence in data interpretation (i.e., interpreting the exosuit as the cause of differences in the observed effects).

## 3. Results

Seven of eight participants increased their lifting repetitions while wearing the exosuit. In case series 1, three of four participants increased their lifting repetitions, ranging from 62 to 75% (68% average, *N* = 3, Table A1). The one remaining participant performed 37% fewer lifts when wearing the exosuit. In case series 2, all four participants increased their lifting repetitions. The magnitude of increase ranged from 8 to 53% (29% average, Table A1) relative to the first control set (A_1_, no exosuit) and from 23 to 50% (38% average, Table A1) relative to the second control set (A_2_, no exosuit). In other words, we confirmed the reversal effect: each participant completed more lifting repetitions right after the exosuit was donned (i.e., B versus A_1_) and then completed fewer lifting repetitions right after the exosuit was removed (i.e., A_2_ versus B). When averaging the control sets (A_1_ and A_2_) together, the increase in lifting repetitions while wearing the exosuit was computed to be from 28 to 38% (34% average, Table A1).

All participants exhibited reduced cumulative back damage during lifting sets with the exosuit relative to sets without the exosuit. This result was found despite seven of eight participants performing more lifting repetitions during the exosuit set.

In case series 1, cumulative damage during the exosuit set was 27–38% less (32% average, Table A2) than cumulative damage during the no exosuit set for the three participants who increased their lifting repetitions. For the remaining participant, his cumulative damage was 65% lower in the exosuit set. The exo moment (defined here as the lumbar extension moment provided by the exosuit at the time of peak lumbar moment, Appendix B) in case series 1 ranged from 15 to 26 Nm (22 Nm average, Table A2).

**Figure 2:**
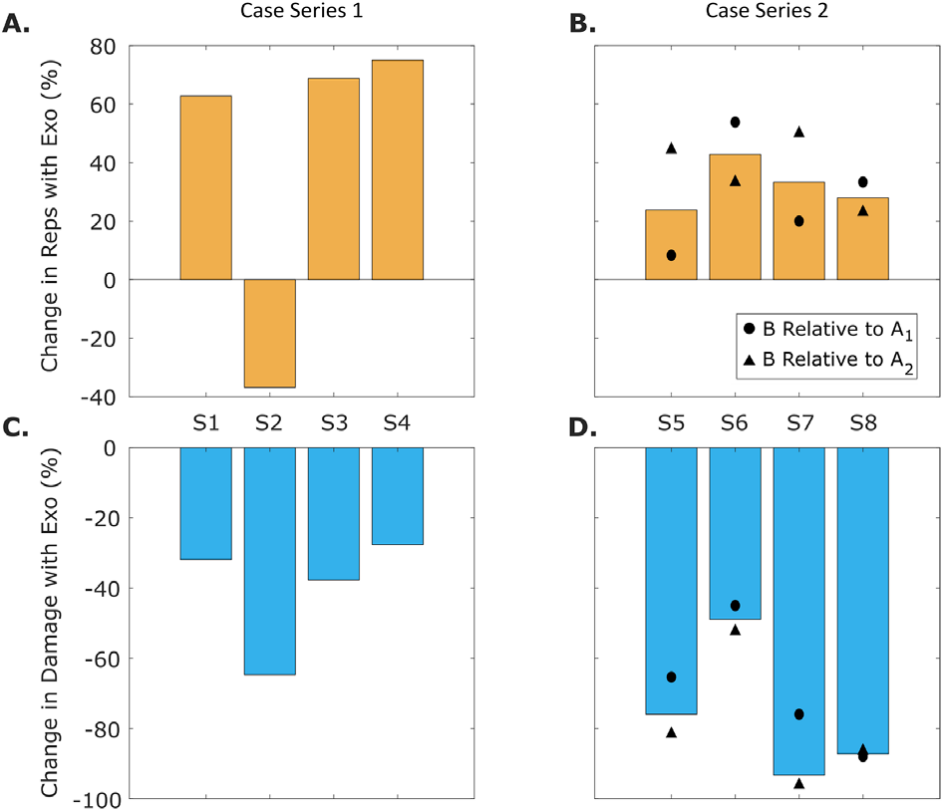
(A, B) Bar plots show the percent change in lifting repetitions when wearing the exosuit relative to not wearing one. Across case series 1 and 2, seven (S1, S3–S8) out of the eight participants increased the number of lifting repetitions they performed while wearing the exosuit. (C, D) Bar plots show the percent change in cumulative damage when wearing the exosuit relative to not wearing one. For example, a value of −40 in this plot represents a 40% reduction in damage when wearing the exosuit relative to not wearing one. Across case series 1 and 2, all eight participants (S1–S8) exhibited lower cumulative damage while wearing the exosuit, despite seven of eight increasing the number of lifting repetitions they performed. In subplots (B) and (D), the bar plots depict results from the exosuit (set B in Figure 1) relative to the average of the two control sets (A_1_, A_2_), while the circle and triangle symbols show comparisons relative to each individual control set (B versus A_1_, and B versus A_2_).

In case series 2, cumulative damage during the exosuit set was 49–93% lower (76% average), when averaging the control sets together (Figure 2). For all participants, cumulative damage decreased after the exosuit was donned (i.e., B versus A_1_), and then cumulative damage increased when the exosuit was doffed (i.e., A_2_ versus B, Figure 2). The exo moment in case series 2 ranged from 27 to 42 Nm (35 Nm average, Table A2). The exo moment magnitude was larger in case series 2 because the participants bent down further to lift this artillery box (relative to the artillery round lifted in case series 1).

**Figure 3.**
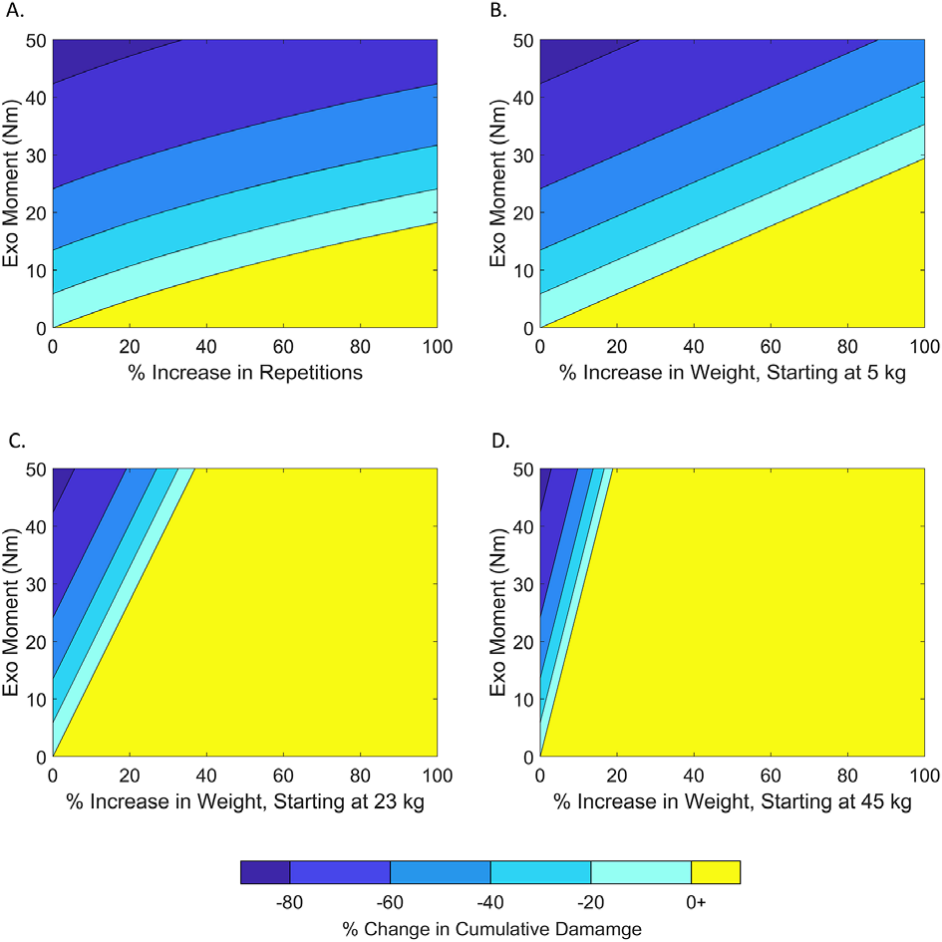
Contour plots show the model-predicted relationships between exo moment, lifting repetitions, object weight, and cumulative damage. Along the *x* axis, a 0% increase represents the nominal lifting scenario, and a 100% increase represents double the weight or repetitions. Negative values shown in shades of blue indicate conditions where there is a decrease in the cumulative damage when wearing an exo relative to the nominal lifting condition without an exo. These negative regions depict when an exo (with an associated exo moment, *y*-axis) could simultaneously increase performance (lifting repetitions or object weight) and reduce risk (cumulative damage). In contrast, yellow regions (0+) of each contour plot indicate conditions when the injury risk benefits of the exo are fully canceled out. For these conditions, there is an increase in the cumulative damage when wearing an exo and increasing lifting repetitions or weight, relative to the nominal lifting condition. Therefore, to receive dual benefits from a back exo (performance enhancement and risk reduction), you want to avoid the yellow regions. (A) The relationship between exo moment, cumulative damage, and lifting repetitions is shown for any constant object weight. Increasing lift repetitions generally does not cancel out reductions in cumulative damage provided by the exo. Next, the relationship between exo moment, cumulative damage, and object weight is shown for nominal object weights of (B) 5 kg, (C) 23 kg, and (D) 45 kg, respectively. The higher the nominal object weight, the smaller the increase (percentage-wise) in the weight needed to cancel out the reduction in cumulative damage provided by the exo.

Parameter sweep models quantified the relationships between exo assistance (lumbar extension moment), lifting repetitions, object weight, and cumulative damage (Figure 3). For a given exo moment, the largest decrease in cumulative damage corresponded to a 0% increase in performance. However, performing more lifting repetitions generally did not cancel out the reductions in cumulative damage due to the exo moment (i.e., the yellow region in Figure 3A is relatively small). For instance, for an exo providing 15.3 Nm (which was the lowest peak assistance experienced by any participant in our case series), the lifting repetitions would need to increase by 79% to fully offset the reduction in cumulative damage from the exo. Furthermore, for an exo providing 42.3 Nm (the highest assistance in the case series), lifting repetitions would need to increase by 399% to fully offset the reduction in cumulative damage from the exo. In contrast, modest increases in object weight were often found to cancel out the exo moment benefits, particularly when considering increasing the weight of objects that are already moderate to heavy weight (Fig. 3B–D). For example, if an exo providing 15.3 Nm of peak assistance was being used to lift a 45-kg object (as in case series 1), then just a 6% (3 kg) increase in object weight would fully offset the exo’s benefits with respect to cumulative damage reduction.

## 4. Discussion

We found that participants wearing a passive elastic back exosuit increased their lifting endurance without canceling out the injury risk benefits of the device. The results of this study indicate that back exos have the potential to simultaneously increase a wearer’s physical capacity to perform lifting work and decrease their low back disorder risks. To achieve these simultaneous benefits, it is preferable to increase lifting repetitions, not object weight. These results have potential implications for back exo users and use cases in defense and civilian sectors.

This was the first study to evaluate the impact of passive elastic back exosuits on lifting endurance when handling heavy objects (>20 kg). Participants in this study increased their lifting endurance by 28–75% when repeatedly lifting heavy objects (45–55 kg). The withdrawal study design (ABA) in case series 2 provided evidence that these improvements in lifting endurance were due to the intervention (i.e., the exosuit), as opposed to random chance, ordering effects, or other factors. These endurance increases were qualitatively similar to but generally higher than two prior studies that found 11–30% increases in endurance when lifting 6–20 kg (Tan et al. 2019; Baltrusch et al. 2020). The observed increases in lifting endurance are further supported by other biomechanics studies that found that back exos can decrease muscle fatigue, metabolic cost, and perceived effort to lift (Kermavnar et al. 2021). It is unclear why a couple of prior studies on different types of back exos found that those specific devices did not increase lifting endurance (Kozinc et al. 2021; So et al. 2022). We speculate that these disparate results in prior studies may have been due to factors such as poor fit, discomfort, less assistance, or more hysteresis with other types of exos. Our biomechanical expectation is that exo extension moments about the low back and hips should augment lifting ability and enhance endurance. This phenomenon is well documented in powerlifting, both in the scientific literature (Blatnik et al. 2012) and in higher world records when lifting with versus without a squat suit, which is essentially an exosuit for powerlifting (Risley 2023).

Although seven of eight participants increased their lifting endurance, one participant performed fewer lifting repetitions when wearing the exosuit. The reason is unknown, in part, because case series 1 only involved an AB test, which makes interpretation more difficult. But we can speculate about a few potential explanations. It may have been because the participant needed more rest time and was still fatigued from their control lifting set, which was performed before the exosuit set. It may have been that this individual required more time to acclimate to the exosuit’s assistance or was just less motivated during the second set. We also observed that this participant received the least amount of assistance from the exosuit out of all the participants (Table A2), suggesting the device may not have been optimally fitted or sized for this particular user. It would have been interesting to recollect the participants in case series 1 using an ABA protocol. However, this was not possible due to the limitations of testing with an active military unit. A limited number of Soldiers were available to participate in this study, we had limited occasions to collect data on base, and we were unable to recruit or ensure the same participants in subsequent visits to the base.

**Figure 4.**
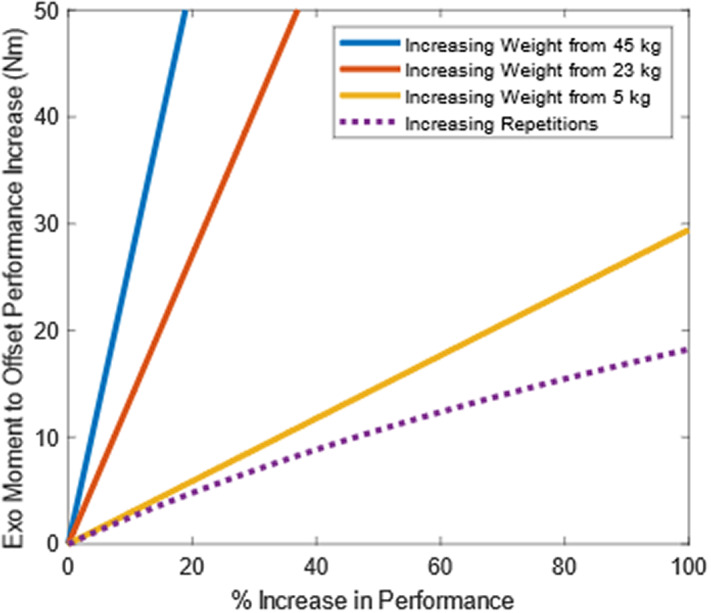
Breakeven contours are shown for the four-parameter sweeps depicted in Figure 3. Each curve represents the conditions at which the cumulative damage reduction benefits of an exo with a given exo moment (*y* axis, in Nm) are perfectly canceled out by an increase in performance (*x* axis). In other words, these curves show conditions of 0% change in cumulative damage relative to the nominal lifting condition without an exo. The curve depends on whether performance increases come from increasing lifting repetitions (purple dotted line) or from increasing object weight from a nominal weight (blue, red, and gold solid lines). Model results indicate that it is preferable to increase performance by increasing the lifting repetitions, not object weight. Exo moment must increase as object weight and lifting repetitions increase to achieve zero change in cumulative damage. For example, a 20% increase in object weight requires 6, 27, and 53 Nm of exo assistance for 5, 23, and 45 kg objects, respectively. However, an increase in lifting repetitions of 20% only needs 5 Nm of exo assistance to maintain constant cumulative damage, regardless of nominal object weight.

Participants incurred less cumulative damage to their low back when wearing the exosuit, even when they performed more lifts. More lifting repetitions will eventually cancel out the injury risk reduction benefits of an exo; however, the model results indicate that for common levels of exo assistance this would generally require an exo to increase lifting repetitions far beyond what was measured in this study (a maximum of 75% increase in lifting repetitions, Figure 2). Cumulative damage increases *linearly* with more lifting repetitions but increases *exponentially* with higher peak loads (Gallagher et al. 2017), which is why it is so impactful to decrease peak musculoskeletal loading with an exo. Our simulation results confirmed that these takeaways are generalizable across material handling work involving heavy lifts, light lifts, high repetitions, and low repetitions (Figures 3 and 4).

**Figure 5.**
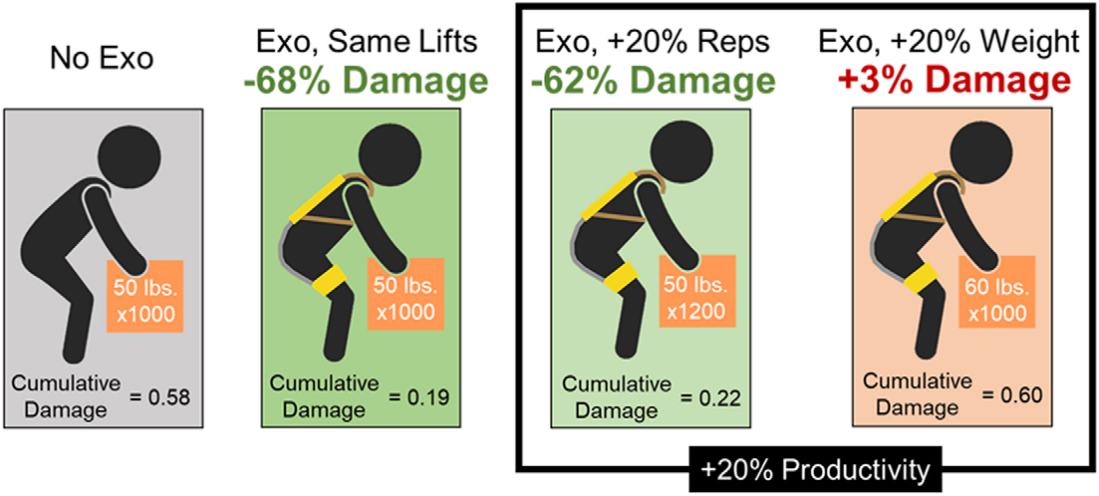
Model results demonstrate why it is preferable to increase lifting repetitions, not object weight, when wearing a back exo. Here are four modeled scenarios: (A) a nominal lifting task: 50 lbs (22.7 kg) lifted 1000 times causes a certain amount of cumulative damage to the back. (B) Completing the same lifting task (i.e., same productivity) with the exo decreased cumulative damage to the back by 68%. The next two modeled tasks involve 20% more productivity (more total weight lifted) than the nominal task. (C) Wearing an exo while also increasing repetitions by 20% leads to a 62% reduction in cumulative damage relative to the nominal task. This is an example of simultaneously increasing productivity and decreasing low back disorder risk. (D) Wearing the exo and increasing object weight by 20% leads to a 3% increase in cumulative damage relative to the nominal task. These modeled scenarios used a 70 cm horizontal distance between the object and spine and a 30 Nm exo moment.

Modeling results indicate that it is generally preferable to increase lifting repetitions, not object weight if using a back exo to increase productivity (e.g., amount of material lifted, loaded, or unloaded). Using an exo to increase lifting repetitions by a given percentage (e.g., by 10%) results in less cumulative damage than increasing object weight by the same percentage (10%). While both increases (in repetitions or weight) can have the same overall effect on lifting productivity or output, they tend to have vastly different effects on musculoskeletal risk metrics like cumulative damage (Figure 5). This conclusion is true for most lifting tasks (e.g., involving moderate or heavy objects) and for exos that provide assistive moments within a typical range for commercially available back exos (e.g., 10–50 Nm) (Di Natali et al. 2021; Madinei et al. 2022; Kang and Mirka 2023). There are certain edge cases, such as when lifting very light weights, but these are generally not relevant to occupational exo use cases. For instance, for very light weights (e.g., less than 5 kg) there are situations where it would technically be more beneficial to increase weight by a given percentage than to increase lift repetitions by that percentage. But these edge cases are often irrelevant in the workplace (e.g., increasing a 1-kg object by 100% to 2 kg) and are typically not the tasks driving worker fatigue or musculoskeletal disorder risks in material handling jobs.

Based on these results, it would be ill-advised to use back exos (such as those tested and modeled in this study) to try to convert a two-person heavy lift (e.g., 80 kg) into a one-person lift, which has been mentioned as a potential capability of interest for the military (Farris et al. 2022). There are some wearable robotic systems that are more akin to wearable forklifts, being developed for this purpose (e.g., Bogue 2018), but this is not the function or capability of most back exos. In general, back exos might provide some limited ability to increase object weights or to enable more individuals to meet physical work requirements by augmenting their strength; however, this requires more research. Based on evidence from this and prior studies (Baltrusch et al. 2018), we advise caution and, in general, do not recommend using most occupational back exos for the purpose of having workers lift substantially heavier weights. Rather, the results of this study support the use of back exos to simultaneously enhance endurance (e.g., lifting repetitions) and reduce musculoskeletal disorder risk (e.g., back overexertion).

The empirical and modeling results from this study indicate that back exos have the potential to simultaneously increase productivity and safety, which is relevant to various defense and civilian jobs. For instance, the field artillery Soldiers who participated in this study could benefit from both augmented endurance and reduced musculoskeletal injury risk (Mudie et al. 2018; Proud et al. 2022). Back exo assistance has the potential to help keep more Soldiers healthy and active, while also enabling them to sustain their lifting, loading, and unloading tasks longer or to complete them faster (e.g., due to needing fewer rest breaks). Thus, exos could have a meaningful impact on both Soldier readiness and operational performance, though these require empirical validation. Other individuals within the Army and other military branches that frequently perform heavy lifts, like those who work in sustainment or distribution units, may also benefit from back exos.

For civilian workers, we expect the trends of less cumulative back damage (Figures 2C–D, 3, and 4) and more lifting endurance with an exosuit (Figure 2A and B) to be similar to the trends we observed in this study with Soldiers. In pilot testing, we had one civilian participant complete a similar lifting protocol but with five sets (ABABA) instead of three (ABA). We found that this individual consistently performed about 58% more lifting repetitions with versus without the exosuit (A_1_: 53, B_1_: 85, A_2_: 53, B_2_: 83, A_3_: 53 lifts). These endurance results further corroborate the trends observed with Soldiers (Figure 2). We also note that the average endurance change in this study (38% increase in lifting repetitions when wearing the exosuit, *N* = 8) is qualitatively consistent with worker-reported results from multi-week field studies in which civilian workers used a similar elastic back exosuit (32% reduction in effort required for their heaviest lifts when wearing the exosuit [SAIF Learning Launch 2021; Nicholson 2022]).

Evidence indicating that exos can have simultaneous productivity and injury risk benefits may help to broaden or solidify the value proposition of occupational exos, as well as ensure exos are being implemented responsibly (i.e., not to increase productivity at the expense of injury risk). Workers wearing an exo who experience less back strain and more endurance might do more lifting, loading, or unloading work during their day. This might increase overall productivity in jobs where lifting (or bending) endurance is a limiting factor, like logistics or agriculture. Increased productivity may provide a shorter-term return on investment from exos in the workplace, which may then complement and enable longer-term benefits, such as fewer injuries or higher worker retention. Ergonomic assessment tools such as Exo-LiFFT could be used to evaluate or make projections about exo effects in conjunction with actual or potential changes in productivity and task demands (e.g., object weight).

In the experimental tests, we observed increases in physical capacity (lifting endurance) when wearing an exosuit, but this outcome is not synonymous with productivity. Higher physical capacity enables the possibility of higher productivity but does not guarantee it. There may be other parts of the body (e.g., arms and hands) or other factors (e.g., psychophysical and operational) that also impose limits on productivity. Furthermore, material handling jobs involve more than just lifting. Most non-lifting parts of a job are not augmented by a back exo. Thus, the productivity gains related to an entire job task (e.g., lifting, carrying, and placing an object, then walking back to get another) are likely to be smaller than the pure lifting endurance benefits measured in this study (28–75%). Indeed, one recent industry study reported an 8% increase in productivity (cases picked per hour) among warehouse workers when wearing an elastic back exosuit similar to the one tested in this study (HeroWear 2024). Additional studies are needed to measure the impact of back exos on productivity and other operational performance outcomes across different jobs and industries.

To assess changes in low back disorder risk, with versus without the exosuit, we used one set of equations based on fatigue failure principles; however, there are other equations or models that could also be used. Here, we used the relationship between cumulative damage and force that is encoded in LiFFT (and Exo-LiFFT) because this is a low back disorder risk assessment tool that has been validated against two epidemiological databases (Gallagher et al. 2017). Alternatively, we could have used established relationships between tissue damage and peak load. For various tissues, cumulative damage has been found to be proportional to peak force to the *C* exponent (i.e., force*^C^*) (Edwards 2018), where *C* is a tissue-specific constant found experimentally via mechanical fatigue studies and generally ranges from 4 to 9 (Carter et al. 1981; Carter and Caler 1985; Thornton et al. 2007; Firminger and Edwards 2021). This indicates that, for example, if an exo reduced peak back loading by 10% then this would be expected to reduce cumulative damage by 34–61%. In effect, in this example, a person wearing an exo would need to increase their lifting repetitions by this same amount (34–61%) to fully cancel out the risk reduction benefits. In summary, even if we had used a different relationship between force and damage, or some other low back disorder risk metric, we believe we would have reached the same general conclusion that it is feasible for an exo to simultaneously enhance lifting endurance and reduce risk, within certain bounds.

There are several limitations to the study worth acknowledging. There were multiple ways we could have measured the impact of the exo on lifting endurance. Some prior studies have assessed endurance using electromyography (Godwin et al. 2009; Lotz et al. 2009; Lamers et al. 2020); however, this approach only evaluates localized (back) muscle fatigue. Other studies have measured task performance outcomes, such as the maximum number of lifts until failure or the maximum number of lifts completed within a specified time limit. After pilot testing a few different protocols, we decided to have participants lift repeatedly until failure. We found this offered a reasonably controlled and consistent way to evaluate lifting endurance. Furthermore, this evaluation had more direct operational relevance to the Army, since these Soldiers frequently complete extended periods of heavy lifting. Lifting until failure provides insight into how long an individual may be able to sustain repetitive lifting work before needing a break. The sample size was relatively small because of the unique challenges of testing with an active military unit. A limited number of Soldiers were available to participate. They had limited training and acclimation time to the exo prototype tested in this study. And we had limited occasions to collect data on base, meaning that at the beginning of the study it was difficult to predict what sample size would be attainable. To account for the likely small sample size, we designed our experiment as a series of case studies. Collectively, the study design along with converging results from this study and industry reports (detailed earlier in the Discussion) are what give us confidence in the conclusion that, at least for a subset of individuals, elastic back exosuits can increase lifting endurance. While the endurance benefits measured empirically are specific to the type of elastic exosuit tested in this study, the ergonomic modeling results are expected to be broadly generalizable to other back exos (e.g., soft, rigid, passive, and powered).

Participants had limited rest time in between lifting sets (20–30 min); however, this did not appear to limit study interpretations or overall conclusions. We acknowledge that the rest time between lifting sets was likely not enough for the participants to recover fully physically (Frey et al. 2022). Nevertheless, the withdrawal (ABA) study design in case series 2 gives us confidence in the conclusion that the exosuit intervention was the reason for the increased endurance (lifting repetitions). Specifically, we found that the lifting repetition results for the A_1_ and A_2_ sets without the exosuit were similar (14 and 13 lifts, on average, Table 1), which would not have occurred if insufficient rest time and fatigue were major confounds. Whereas lifting repetitions increased when wearing the exosuit (18 lifts, on average, Table 1). Notably, in case series 2, all four participants increased their endurance right after the exosuit was donned (B versus A_1_, Fig. 2B) and then exhibited decreased endurance right after the exosuit was removed (A_2_ versus B, Fig. 2B). In case series 1, we chose to always test the exosuit second to conservatively estimate the increase in lifting endurance the exosuit provided. If participants were given additional time to recover, or if the testing order had been randomized, then the exosuit’s endurance benefits may have been even larger than measured in this study. At present, the study results suggest that, at least for a subset of individuals, there is reliable and repeatable evidence that the exosuit was capable of enhancing lifting endurance. Participants also had limited acclimation time (10–20 min), but this was still sufficient to demonstrate the exosuit’s benefits in this study and prior studies (e.g., Yandell et al. 2022; Slaughter et al. 2023). However, extended acclimation time has been found to improve the benefits a user gets from an exo (Poggensee and Collins 2021). In view of these study limitations, it is worth acknowledging that lifting endurance benefits from the exosuit in this study may increase with more acclimation or more rest between test sets.

The study only tested young men due to the demographics of U.S. Army field artillery units (>90% men) and volunteers for this study. In addition, this study was limited to heavy lifting; however, we anticipate that exosuits may increase endurance even more when lifting lighter weights because the exo moment contributes a higher percentage of the total lumbar moment. Because back exosuits are wearable devices that exert forces on participants, it was not feasible to blind participants to this intervention. Other psychosocial effects may also have impacted lifting performance, such as occurred in case series 1 with the one excluded participant (see Methods). To limit these potential effects, we staggered participant start times in case series 2. When evaluating risk in this study, we focused solely on low back disorder risk. Cumulative back damage was used as an indicator of risk. Damage was estimated based on empirical relationships observed between peak tissue force and fatigue failure (Gallagher et al. 2017). We did not directly measure physiological tissue damage, nor did we evaluate risk to other parts of the body like the shoulders, hips, or knees.

## 5. Conclusions

In summary, we found evidence that individuals who wore passive elastic back exosuits increased their physical capacity (lifting endurance) without canceling out the musculoskeletal risk reduction benefits from the exosuit. This dual benefit can be achieved by increasing the number of lifting repetitions performed while wearing an exo, which is generally preferable to increasing object weight. These results have important implications for the occupational safety and health field and for workers in civilian and defense sectors, suggesting back exos may make it possible to simultaneously boost productivity and reduce work-related musculoskeletal disorders.

## Supporting information

Rodzak et al. supplementary material Rodzak et al. supplementary material

## Data Availability

Data presented will be provided by the corresponding author upon request.

## Supplementary material

The supplementary material for this article can be found at http://doi.org/10.1017/wtc.2024.8.

## References

[r1] Annual Injury Surveillance Report 2016 Summary (Annual No. 12-110–0520) (2016). U.S. Army Public Health Center. https://ph.health.mil/Periodical_Library/USArmyInjurySurveillanceSummary2016.pdf (accessed July 12 2022).

[r2] Annual Injury Surveillance Report 2017 Summary (Annual No. 12-109–0520) (2017). U.S. Army Public Health Center. https://ph.health.mil/Periodical_Library/USArmyInjurySurveillanceSummary2017.pdf (accessed July 12 2022).

[r3] Annual Injury Surveillance Report 2018 Summary (Annual No. 12-113–0820) (2018). U.S. Army Public Health Center. https://ph.health.mil/Periodical_Library/USArmyInjurySurveillanceSummary2018.pdf (accessed July 12 2022).

[r4] Annual Injury Surveillance Report 2019 Summary (Annual No. 12-114–0121) (2019). U.S. Army Public Health Center. https://apps.dtic.mil/sti/trecms/pdf/AD1136242.pdf (accessed July 12 2022).

[r5] Baltrusch SJ, van Dieen JH, van Bennekom CAM and Houdijk H (2020) Testing an exoskeleton that helps workers with low-Back pain: Less discomfort with the passive SPEXOR trunk device. IEEE Robotics & Automation Magazine 27, 66–76. 10.1109/MRA.2019.2954160

[r6] Baltrusch SJ, van Dieën JH, van Bennekom CAM and Houdijk H (2018) The effect of a passive trunk exoskeleton on functional performance in healthy individuals. Applied Ergonomics 72, 94–106. 10.1016/j.apergo.2018.04.007 29885731

[r7] Bensel CK, Corner B, Gregorczyk KN, Schiffman JM (2008) Understanding the physiological, biomechanical, and performance effects of body armor use 10. https://www.researchgate.net/profile/Carolyn-Bensel/publication/235200699_Understanding_the_Physiological_Biomechanical_and_Performance_Effects_of_Body_Armor_Use/links/00b7d52cd65e423786000000/Understanding-the-Physiological-Biomechanical-and-Performance-Effects-of-Body-Armor-Use.pdf

[r8] Blatnik JA, Skinner JW and McBride JM (2012) Effect of supportive equipment on force, velocity, and power in the squat. Journal of Strength and Conditioning Research 26, 3204–3208. 10.1519/JSC.0b013e3182736641 22996018

[r9] Bogue R (2018) Exoskeletons – a review of industrial applications. IR 45, 585–590. 10.1108/IR-05-2018-0109

[r10] Brinckmann P, Biggemann M, and Hilweg D (1988) Fatigue fracture of human lumbar vertebrae. Clinical Biomechanics 3, i-S23. 10.1016/S0268-0033(88)80001-9 23905925

[r11] Bureau of Labor Statistics (2020) Case circumstances and worker characteristics for injuries and illnesses involving days away from work – MSD by part of body affected by days away from work. Case circumstances and worker characteristics for injuries and illnesses involving days away from work. Survey of Occupational Injuries and Illnesses Data.

[r12] Carter DR and Caler WE (1985) A cumulative damage model for bone fracture. Journal of Orthopaedic Research 3, 84–90. 10.1002/jor.1100030110 3981298

[r13] Carter DR, Caler WE, Spengler DM and Frankel VH (1981) Fatigue behavior of adult cortical bone: the influence of mean strain and strain range. Acta Orthopaedica Scandinavica 52, 481–490. 10.3109/17453678108992136 7331784

[r14] Chang SE, Pesek T, Pote TR, Hull J, Geissinger J, Simon AA, Alemi MM and Asbeck AT (2020) Design and preliminary evaluation of a flexible exoskeleton to assist with lifting. Wearable Technologies 1, e10. 10.1017/wtc.2020.10 39050263 PMC11264825

[r15] Di Natali C, Chini G, Toxiri S, Monica L, Anastasi S, Draicchio F, Caldwell D and Ortiz J (2021) Equivalent weight: Connecting exoskeleton effectiveness with ergonomic risk during manual material handling. IJERPH 18, 2677. 10.3390/ijerph18052677 33799947 PMC7967312

[r16] dos Anjos FV, Ghislieri M, Cerone GL, Pinto TP and Gazzoni M (2022) Changes in the distribution of muscle activity when using a passive trunk exoskeleton depend on the type of working task: A high-density surface EMG study. Journal of Biomechanics 130, 110846. 10.1016/j.jbiomech.2021.110846 34749163

[r17] Edwards WB (2018) Modeling overuse injuries in sport as a mechanical fatigue phenomenon. Exercise and Sport Sciences Reviews 46, 224–231. 10.1249/JES.0000000000000163 30001271

[r18] Farris DJ, Harris DJ, Rice HM, Campbell J, Weare A, Risius D, Armstrong N and Rayson MP (2022) A systematic literature review of evidence for the use of assistive exoskeletons in defence and security use cases. Ergonomics, 1–27. 10.1080/00140139.2022.2059106 35348442

[r19] Firminger CR and Edwards WB (2021) Effects of cyclic loading on the mechanical properties and failure of human patellar tendon. Journal of Biomechanics 120, 110345. 10.1016/j.jbiomech.2021.110345 33735631

[r20] Fox S, Aranko O, Heilala J and Vahala P (2019) Exoskeletons: Comprehensive, comparative and critical analyses of their potential to improve manufacturing performance. JMTM 31, 1261–1280. 10.1108/JMTM-01-2019-0023

[r21] Frey MF, Howarth S and De Carvalho D (2022) Does a study protocol that includes two trunk endurance tests risk carry-over effects? – A pilot study. Presented at the North American Congress of Biomechanics, Ottawa, Canada.

[r22] Gallagher S and Barbe MF (2022) Musculoskeletal Disorders: Causes and Control, 1st Edn. Hoboken, NJ: John Wiley & Sons, Inc.

[r23] Gallagher S and Heberger JR (2015) Examining the interaction of force and repetition on musculoskeletal disorder risk: a systematic literature review. Human Factors, 55(1), 108–124. 10.1177/0018720812449648 PMC4495348 23516797

[r24] Gallagher S, Sesek RF, Schall MC and Huangfu R (2017) Development and validation of an easy-to-use risk assessment tool for cumulative low back loading: The lifting fatigue failure tool (LiFFT). Applied Ergonomics 63, 142–150. 10.1016/j.apergo.2017.04.016 28477843

[r25] Genaidy A and Asfour S (1989) Effects of frequency and load of lift on endurance time. Ergonomics 32, 51–57. 10.1080/00140138908966067 2924761

[r26] Godwin AA, Stevenson JM, Agnew MJ, Twiddy AL, Abdoli-Eramaki M and Lotz CA (2009) Testing the efficacy of an ergonomic lifting aid at diminishing muscular fatigue in women over a prolonged period of lifting. International Journal of Industrial Ergonomics 39, 121–126. 10.1016/j.ergon.2008.05.008

[r27] Golabchi A, Chao A and Tavakoli M (2022) A systematic review of industrial exoskeletons for injury prevention: Efficacy evaluation metrics, target tasks, and supported body postures. Sensors 22, 2714. 10.3390/s22072714 35408328 PMC9002381

[r28] Goršič M, Song Y, Dai B and Novak D (2021) Evaluation of the HeroWear Apex back-assist exosuit during multiple brief tasks. Journal of Biomechanics 126, 110620. 10.1016/j.jbiomech.2021.110620 34293602 PMC8453127

[r29] Gun BK, Banaag A, Khan M and Koehlmoos TP (2022) Prevalence and risk factors for musculoskeletal Back injury among U.S. Army personnel. Military Medicine 187, e814–e820. 10.1093/milmed/usab217 34159385

[r60] HeroWear: (2020) Back-assist Wearable Tech for Men & Women | Exoskeleton Technology From HeroWear [WWW Document]. https://herowearexo.com/ (accessed 7.26.22).

[r30] HeroWear (2024) International grocery retailer improves worker well-being, boosts productivity with exosuits. https://herowearexo.com/wp-content/uploads/HeroWear-Case_Study-Productivity-Worker-Well-Being-2024.pdf (accessed February 7 2024).

[r31] Hollander IE and Bell NS (2010) Physically demanding jobs and occupational injury and disability in the U.S. Army. Military Medicine 175, 705–712. 10.7205/MILMED-D-09-00214 20968258

[r32] Kang SH and Mirka GA (2023) Effect of trunk flexion angle and time on lumbar and abdominal muscle activity while wearing a passive back-support exosuit device during simple posture-maintenance tasks. Ergonomics, 1–11. 10.1080/00140139.2023.2191908 36921063

[r33] Kermavnar T, de Vries AW, de Looze MP and O’Sullivan LW (2021) Effects of industrial back-support exoskeletons on body loading and user experience: An updated systematic review. Ergonomics 64, 685–711. 10.1080/00140139.2020.1870162 33369518

[r34] Kozinc Ž, Baltrusch S, Houdijk H and Šarabon N (2021) Short-term effects of a passive spinal exoskeleton on functional performance, discomfort and user satisfaction in patients with low Back pain. Journal of Occupational Rehabilitation 31, 142–152. 10.1007/s10926-020-09899-7 32356222

[r35] Lamers E, Zelik K and Yang A (2018) Feasibility of a biomechanically-assistive garment to reduce low Back loading during leaning and lifting. IEEE Transactions on Biomedical Engineering 65, 1674–1680. 10.1109/TBME.2017.2761455 28991732 PMC8820216

[r36] Lamers EP, Soltys JC, Scherpereel KL, Yang AJ and Zelik KE (2020) Low-profile elastic exosuit reduces back muscle fatigue. Scientific Reports 10, 15958. 10.1038/s41598-020-72531-4 32994427 PMC7524767

[r37] Lamers EP and Zelik KE (2021) Design, modeling, and demonstration of a new dual-mode back-assist exosuit with extension mechanism. Wearable Technologies 2, e1. 10.1017/wtc.2021.1 36325150 PMC9624433

[r38] Liebl H, Schinz D, Sekuboyina A, Malagutti L, Löffler MT, Bayat A, El Husseini M, Tetteh G, Grau K, Niederreiter E, Baum T, Wiestler B, Menze B, Braren R, Zimmer C and Kirschke JS (2021) A computed tomography vertebral segmentation dataset with anatomical variations and multi-vendor scanner data. Scientific Data 8, 284. 10.1038/s41597-021-01060-0 34711848 PMC8553749

[r39] Löffler MT, Sekuboyina A, Jacob A, Grau A-L, Scharr A, El Husseini M, Kallweit M, Zimmer C, Baum T and Kirschke JS (2020) A vertebral segmentation dataset with fracture grading. Radiology: Artificial Intelligence 2, e190138. 10.1148/ryai.2020190138 33937831 PMC8082364

[r40] Lotz CA, Agnew MJ, Godwin AA and Stevenson JM (2009) The effect of an on-body personal lift assist device (PLAD) on fatigue during a repetitive lifting task. Journal of Electromyography and Kinesiology 19, 331–340. 10.1016/j.jelekin.2007.08.006 18055220

[r41] Madinei S, Kim S, Park J-H, Srinivasan D and Nussbaum MA (2022) A novel approach to quantify the assistive torque profiles generated by passive back-support exoskeletons. Journal of Biomechanics 145, 111363. 10.1016/j.jbiomech.2022.111363 36332510

[r42] Mudie KL, Boynton AC, Karakolis T, O’Donovan MP, Kanagaki GB, Crowell HP, Begg RK, LaFiandra ME and Billing DC (2018) Consensus paper on testing and evaluation of military exoskeletons for the dismounted combatant. Journal of Science and Medicine in Sport 21, 1154–1161. 10.1016/j.jsams.2018.05.016 30318056

[r43] Nicholson P (2022) Safety Innovation Challenge: HeroWear Apex. San Diego, CA: National Safety Council Congress.

[r44] Poggensee KL and Collins SH (2021) How adaptation, training, and customization contribute to benefits from exoskeleton assistance. Science Robotics 14. eabf1078. 10.1126/scirobotics.abf1078 34586837

[r45] Potvin JR and Norman RW (1993) Quantification of erector spinae muscle fatigue during prolonged, dynamic lifting tasks. European Journal of Applied Physiology 67, 554–562. 10.1007/BF00241654 8149937

[r46] Proud JK, Lai DTH, Mudie KL, Carstairs GL, Billing DC, Garofolini A and Begg RK (2022) Exoskeleton application to military manual handling tasks. Human Factors 64, 527–554. 10.1177/0018720820957467 33203237

[r47] Quirk DA, Chung J, Applegate M, Cherin JM, Dalton DM, Awad LN and Walsh CJ (2023) Evaluating adaptiveness of an active back exosuit for dynamic lifting and maximum range of motion. Ergonomics 67, 1–14. 10.1080/00140139.2023.2240044 37482538 PMC10803634

[r48] Reynolds K, Cosio-Lima L, Creedon J, Gregg R and Zigmont T (2002) Injury occurrence and risk factors in construction engineers and combat artillery soldiers. Military Medicine 167, 971–977. 10.1093/milmed/167.12.971 12502169

[r49] Risley K (2023) Powerlifting Records: Raw & Equipped [WWW Document]. Lift Vault. https://liftvault.com/resources/powerlifting-records/ (accessed 5.12.23).

[r50] SAIF Learning Launch: how exosuits can help reduce back strains and sprains (2021). (accessed October 9 2023)

[r51] Schechtman H and Bader DL (1997) In vitro fatigue of human tendons. Journal of Biomechanics 30, 829–835. 10.1016/S0021-9290(97)00033-X 9239568

[r52] Sekuboyina A, Husseini ME, Bayat A, Löffler M, Liebl H, Li H, Tetteh G, Kukačka J, Payer C, Štern D, Urschler M, Chen M, Cheng D, Lessmann N, Hu Y, Wang T, Yang D, Xu D, Ambellan F, Amiranashvili T, Ehlke M, Lamecker H, Lehnert S, Lirio M, Olaguer NPD, Ramm H, Sahu M, Tack A, Zachow S, Jiang T, Ma X, Angerman C, Wang X, Brown K, Kirszenberg A, Puybareau É, Chen D, Bai Y, Rapazzo BH, Yeah T, Zhang A, Xu S, Hou F, He Z, Zeng C, Xiangshang Z, Liming X, Netherton TJ, Mumme RP, Court LE, Huang Z, He C, Wang L-W, Ling SH, Huỳnh LD, Boutry N, Jakubicek R, Chmelik J, Mulay S, Sivaprakasam M, Paetzold JC, Shit S, Ezhov I, Wiestler B, Glocker B, Valentinitsch A, Rempfler M, Menze BH and Kirschke JS (2021) VerSe: A vertebrae labelling and segmentation benchmark for multi-detector CT images. Medical Image Analysis 73, 102166. 10.1016/j.media.2021.102166 34340104

[r53] Slaughter P, Rodzak K, Fine S, Ice C, Wolf D and Zelik K (2023) Evaluation of U.S. Army soldiers wearing a back exosuit during a field training exercise. Wearable Technologies 4. 10.1017/wtc.2023.16. PMC10936316 38487775

[r54] So BCL, Hua C, Chen T, Gao Q and Man SS (2022) Biomechanical assessment of a passive back-support exoskeleton during repetitive lifting and carrying: Muscle activity, kinematics, and physical capacity. Journal of Safety Research 83, 210–222. 10.1016/j.jsr.2022.08.017 36481011

[r55] Tan CK, Kadone H, Miura K, Abe T, Koda M, Yamazaki M, Sankai Y and Suzuki K (2019) Muscle synergies during repetitive stoop lifting with a bioelectrically-controlled lumbar support exoskeleton. Frontiers in Human Neuroscience 13, 142. 10.3389/fnhum.2019.00142 31114492 PMC6503089

[r56] Thornton GM, Schwab TD and Oxland TR (2007) Cyclic loading causes faster rupture and strain rate than static loading in medial collateral ligament at high stress. Clinical biomechanics 22, 932–940. 10.1016/j.clinbiomech.2007.05.004 17602807

[r57] van Harmelen V, Schnieders J, Wagemaker SJ (2022) Measuring the amount of support of lower back exoskeletons. https://static1.squarespace.com/static/5f7d9bb22f1bc82b03f6f1b0/t/6357ace65eff997ad8002040/1670252213534/Laevo+V2.5+and+FLEX+support+torque+measurement+-+white+paper+with+competitor+curves+V03.pdf (accessed December 19 2022)

[r58] Yandell MB, Wolfe AE, Marino MC, Harris MP and Zelik KE (2022) Effect of a Back-assist exosuit on logistics worker perceptions, acceptance, and muscle activity. In Moreno JC, Masood J, Schneider U, Maufroy C and Pons JL (eds), Wearable Robotics: Challenges and Trends, Biosystems & Biorobotics. Cham: Springer International Publishing, pp. 7–11. 10.1007/978-3-030-69547-7_2.

[r59] Zelik KE, Nurse CA, Schall MC, Sesek RF, Marino MC and Gallagher S (2022) An ergonomic assessment tool for evaluating the effect of back exoskeletons on injury risk. Applied Ergonomics 99, 103619. 10.1016/j.apergo.2021.103619 34740072 PMC9827614

