# Supplementary material for: Can back exosuits simultaneously increase lifting endurance and reduce musculoskeletal disorder risk?
Source: Wearable Technol. 2024 Nov 28;5:e17. doi: 10.1017/wtc.2024.8 (PMC11729484; doi:10.1017/wtc.2024.8)
Supplement: Rodzak et al. supplementary material [file S2631717624000082sup001.pdf]

Supplement to *Can back exosuits simultaneously increase lifting endurance and reduce musculoskeletal disorder risk?*

Appendix A

Appendix A provides participant-specific lifting and cumulative damage results for cases series 1 and 2.

Table 1. Participant characteristics.

	Participant	Age	Height (cm)
Case Series 1	1	20	185.4
	2	20	175.3
	3	33	182.9
	4	25	172.7
Case Series 2	5	19	182.9
	6	21	185.4
	7	20	182.9
	8	20	172.7

Table 2. Lifting repetitions completed by each participant with vs. without the exosuit in case series 1 and 2. Seven of eight participants increased their lifting repetitions when wearing the exosuit. Participants lifted 45 kg artillery shells in case series 1 and 55 kg artillery boxes in case series 2. Lifts were performed every 6 seconds until failure.

	Participant	Lifting Repetitions		
		A_1 (No Exo)	B (Exo)	A_2 (No Exo)
Case Series 1	1	51	83	-
	2	38	24	-
	3	16	27	-
	4	48	84	-
	Average (Range)	38.25 (16-51)	54.5 (24-84)	-
Case Series 2	5	12	13	9
	6	13	20	15
	7	20	24	16
	8	12	16	13
	Average (Range)	14.25 (12-20)	18.25 (13-24)	13.25 (9-16)

Table 3. Cumulative damage to the back for each participant with vs. without the exosuit in case series 1 and 2. For all participants, cumulative damage was lower during lifting sets when wearing the exosuit. The exo moment (i.e., lumbar extension moment provided by the exosuit at the time of peak lumbar moment) is also presented in the table. For the purposes of this study, it is simplest to think of damage as a unitless metric and to focus solely on relative differences between the exo vs. no exo conditions. However, in principle, there is an established relationship between tissue damage and cycles to failure, which gives deeper insight into the meaning and units of cumulative damage. To learn more about cumulative damage and fatigue failure principles we refer readers to (Edwards, 2018) and (Gallagher and Barbe, 2022). The orders of magnitude differences in damage between case series 1 and 2 are a result of three things: (i) the non-linear relationship between peak moment and cumulative damage (Gallagher et al., 2017; Zelik et al., 2022), (ii) the larger object-to-spine distance (moment arm) in case series 2, and (iii) the heavier object in case series 2.

	Participant	Exo Moment (Nm)	Cumulative Damage		
			A_1 (No Exo)	B (Exo)	A_2 (No Exo)
Case Series 1	1	22.9	0.09	0.06	-
	2	15.3	0.02	0.01	-
	3	26.2	0.26	0.16	-
	4	23.2	0.11	0.08	-
Case Series 2	5	32.7	692.6	240.0	1301.4
	6	27.1	552.4	303.9	637.4
	7	42.3	203.7	49.0	1254.3
	8	36.4	2609.7	314.6	2305.3

Appendix B

Appendix B details how we estimated the exo moment used to calculate cumulative damage when participants were wearing an exosuit. Broadly speaking, exo moment refers to the time-varying lumbar extension moment generated by a back exo. Assessing the effect of a back exo on low back disorder risk with Exo-LiFFT (an ergonomic assessment tool) nominally requires a single exo moment value to be extracted from the time-series data and used as an input. There are multiple ways to approximate or select this exo moment value, but some are preferable to others. In this study, we estimated the exo moment at the time of peak lumbar moment (i.e., peak loading on the user's back). This exo moment metric is slightly different from—but we believe an improvement upon—the approximation used in the original Exo-LiFFT paper (Zelik et al., 2022), for reasons detailed below.

Motivation & Rationale

It is useful to provide context and rationale for why we extracted exo moment at this instant of peak lumbar moment before explaining how methodologically we performed the estimates. Cumulative damage to the low back can provide an indicator of low back disorder risk in lifting-intensive jobs such as material handling (Gallagher et al., 2017). Cumulative damage is a function of peak back loading (e.g., muscle and spinal forces). Thus, the challenge with back exos is estimating how much they reduce peak loading of a user's back.

In general, back exos do not provide their peak assistive moment at the time of peak back loading (i.e., peak lumbar moment). Thus, one cannot simply extract the peak exo moment reached over the entire lifting cycle and subtract this contribution, because this would tend to overestimate the back offloading and injury risk reduction benefits of exos. The magnitude difference between peak exo moment (over the entire lifting cycle) and exo moment at the time of peak lumbar moment can be minimal or it can be significant, depending on the specific exo. What we originally recommended in Zelik et al. (2022) was to use "peak exo moment at or near the time of peak load moment," which generally corresponds with the deepest part of the lift or the time immediately before an object is lifted. This can be a reasonable approximation of the reduction in back loading for certain types of back exos, particularly when movement biomechanics data are not available. But this approximation is not appropriate for all exos or circumstances. The problem is that the exo moment at this instant in time (immediately before lifting) can sometimes over- or under-estimate how much an exo reduces peak loading on the user's back. This problem is apparent for both passive (elastic) and powered (motorized) exos, for reasons explained below.

For passive exos, peak exo moment tends to precede peak lumbar moment (when overall loading on the person's back is the highest). This is due to a combination of postural effects, hysteresis, and the trunk-worn weight of the exo. Postural effects refer to the fact that peak lumbar moment typically occurs a short time after peak trunk-to-thigh angle, due to the dynamics of accelerating the body and object upwards. Therefore, the user's body tends to be in a slightly less flexed posture, which equates to slightly less displacement (and force and moment) from the elastic element of the exo. Exo hysteresis refers to mechanical energy loss due to friction in the elastic element(s), interface(s) to the body, and underlying biological tissues. Hysteresis results in lower elastic force during the lifting (ascending, extension) phase relative to the lowering (descending, flexion) phase of the movement. The magnitude of hysteresis depends on the specific exo and type

of elastic material or spring mechanism used, and it can be large or small. For instance, Chang et al. (2020) reported that the hysteresis of springs varies based on the material/type from less than 1% to more than 15%. Furthermore, van Harmelen et al. (2022) used a mannequin to evaluate six different elastic back exo products (using gas springs, fiberglass rods, or fabric elastic elements) and reported overall device hysteresis values ranging from 28-51%. These results indicate that for some devices the exo moment at the time of peak lumbar moment can be substantially lower than the peak exo moment over the entire lift. Given the wide range of hysteresis values found for different springs (elastic elements) and exo products, we believe it is prudent to estimate these values for specific exos of interest. Finally, the trunk-worn weight of the exo creates a lumbar flexion moment during forward bending that can partially counteract the lumbar extension moment provided by the exo's elastic element. Thus, trunk-worn weight should also be accounted for when calculating the net exo moment contributions.

For powered exos, peak exo moment tends to occur later than peak lumbar moment (when overall loading on the person's back is the highest). This is largely due to powered actuator and control limitations. The net exo moment is also affected by the trunk-worn weight of the exo. As mentioned above, added trunk weight can partially counteract the lumbar extension moment provided by the exo. Thus, the biomechanical effect of the trunk-worn weight during trunk flexion should be calculated. Powered exos rely on sensors to identify when to exert assistive forces. Exo controllers need to sense movement data over a sufficient window of time to make assistance decisions and avoid too many false positives (e.g., exerting inappropriate or inopportune forces on the user such as when they are not lifting). Thus, there is a time lag between when the user begins their lifting movement and when the exo controller determines that a lift has begun and that it should assist. Furthermore, the powered actuator (e.g., motor) has its own limits in terms of how quickly it can ramp up force due to both user safety and physical hardware limitations.

As an example, Quirk et al. (2023) performed a human subject evaluation of a powered back exosuit during stoop lifting. The peak moment (about the low back) the powered exosuit applied during the lifts was 23.7 Nm (*N*=14). But at the time of peak lumbar moment, the moment from the exosuit motors was 17.3 Nm (27% lower). After accounting for the trunk-worn weight of the powered exosuit (approximately 2.5 kg), the distance from this exo weight to the lumbar spine (about 40 cm), and the trunk flexion during lifting (about 70 degrees), we found that the exo assistance moment was further reduced by 9.2 Nm. Thus, the trunk-worn weight resulted in an exo moment that was much lower than the peak moment generated by the motors. Ultimately, this biomechanical analysis indicates that the powered exosuit exerted about 8.1 Nm of exo moment (i.e., net moment about the low back at the time of peak back loading), which was 66% lower than the peak moment exerted by the exo's motors during the lift. In summary, the low back relief (reduction in back muscle loading) experienced by the wearer was only about one third of the peak assistance output by this powered exosuit.

Collectively, these empirical data on passive and powered back exos exemplify the need to be careful and thoughtful in computing the exo moments that are input into ergonomic risk assessment models. This is necessary to ensure exo assistance is appropriate and indicative of the amount an exo reduces peak loading to the user's back. When possible, we recommend using exo moment at the time of peak lumbar moment as the input to ergonomic assessment models such as Exo-LiFFT. The section below details how specifically we estimated exo moment at the time of peak lumbar moment in this study.

Calculation of Exo Moment at the Time of Peak Lumbar Moment in this Study

First, we used participant-specific data collected during each case series to estimate the peak exosuit force during the lift. For the exosuit evaluated in this study, the peak force is exerted by the elastic bands at the deepest part of the lift. This is when the exosuit's elastic bands are most stretched. We measured the stretch in the elastic bands when participants were at peak trunk-to-thigh angle (i.e., just before lifting). We then used the difference between the stretched and rest lengths of the bands with force-displacement tables provided by the manufacturer of the bands (HeroWear, Nashville, TN, USA) to calculate the peak force in the elastic bands (F_{peak}) during a lift.

Second, we calculated the peak exo moment (M_{peak}), meaning the lumbar extension moment generated by the exosuit's elastic bands at peak trunk-to-thigh angle. This was computed for each case series participant by multiplying the peak force by the moment arm of the elastic bands about the lumbar spine (Equation A1). We estimated this moment arm by summing: (i) the distance from the bands to the skin surface of low back (x_1) with (ii) the distance from the center of the L5/S1 joint to the skin surface (x_2). These distances were roughly perpendicular to the line of action of the exosuit elastic bands. We used a tape measure to measure x_1 at approximately the L5/S1 level for each participant while they were in the deepest part of their lift. We found x_1 to be 0.9 ± 1.0 cm (across both case series, *N*=8). Next, we found a typical L5/S1 to skin distance using CT scans. Specifically, we measured x_2 from the median plane in 109 segmented CT scans from healthy adults (Liebl et al., 2021; Löffler et al., 2020; Sekuboyina et al., 2021) using 3D Slicer software. We measured x_2 to be 9.93 ± 1.2 cm.

$$M_{peak} = F_{peak}(x_1 + x_2) \qquad \text{Equation A1}$$

Third, we used peak exo moment to estimate the exo extension moment at the time of peak lumbar moment (M_{ext}) using a relationship we derived in a separate experiment. We performed a motion analysis lab case study (*N*=1) to estimate the magnitude of exo moment at peak lumbar moment in relation to the peak exo moment (i.e., at peak trunk-to-thigh flexion). A male participant was consented under a protocol approved by the Vanderbilt University Institutional Review Board. He wore a SABER exosuit during twenty repeated lifts of a 16 kg dumbbell off of the floor. Ten of the lifts were performed using a squat technique and ten were performed using a stoop technique. During the lifting, we measured lower body and trunk kinematics using an optical motion capture system (Vicon, Oxford, UK) and ground reaction forces via in-ground force plates (AMTI, Watertown, MA, USA). Additionally, we placed a load cell (Futek, Irvine, CA, USA) above and in line with the elastic bands to directly measure the force through the bands.

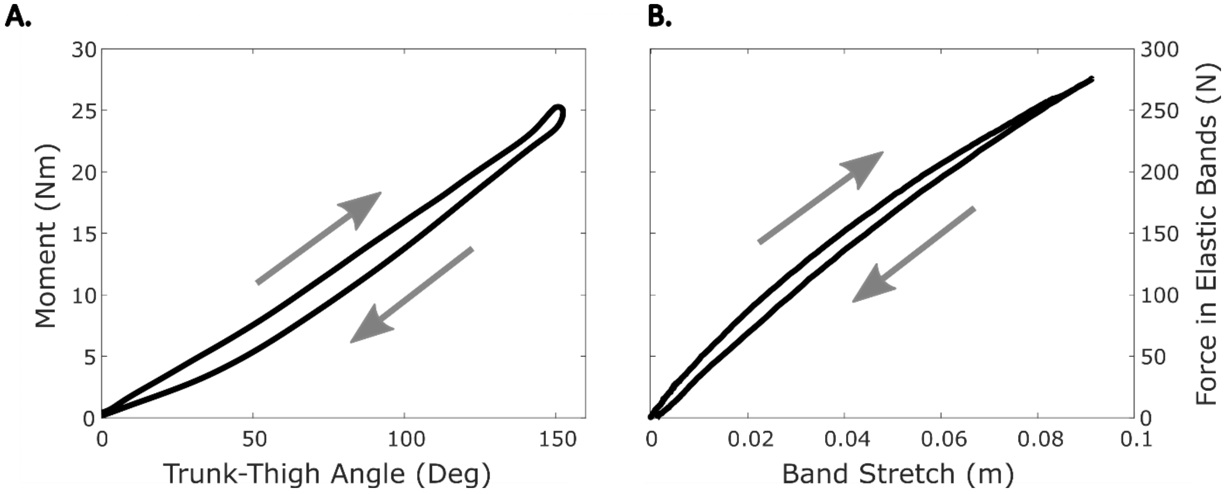

Figure A1. Work loops for the (A) exosuit when worn by a user, and (B) elastic bands alone. A) Exosuit on a user: The force in the exosuit's elastic bands is plotted as a function of the participant's trunk-to-thigh angle. We found hysteresis of the entire exosuit system to be 12 ± 3% based on the kinematic and load cell data collected in this case study. This hysteresis estimate was computed as the integral of the work loop, signifying the percentage of energy lost over the entire loading (upward arrow) and unloading (downward arrow) cycle. It includes energy losses from the elastic bands, physical interfaces to the body, and underlying biological tissues. (B) Isolated elastic bands: Elastic band force is plotted as a function of band stretch. These data were collected when stretching the elastic bands (separate from the exosuit or user) by using a load cell in series with the elastic bands and optical motion capture to measure band length changes. We found the hysteresis of the elastic bands to be 7 ± 2%. The specific elastic band used was the S2000 Size 2 elastic band (HeroWear, Nashville, TN, USA) which was the most common band size used in case series 1 and 2. The lefthand axis is based on exo moment estimates for the case study participant in the Appendix, whose exo moment arm was estimated to be 10 cm.

For each lift, we calculated the lumbar moment about the L5/S1 joint using bottom-up inverse dynamics, which includes the weight of the exosuit in the calculation of the lumbar moment. We then found the time and magnitude of the peak lumbar moment. We also calculated the trunk-to-thigh angle and found the timing of the peak, which we defined as the deepest point of the lift. We computed the time-series exo moment by multiplying the force measured by the load cell with a 10 cm moment arm ($x_1 + x_2$ for this case study participant). We extracted the exo moment (i) at the deepest point in the lift (M_{peak}), and (ii) at the time of peak lumbar moment (M_{ext}, Fig. A2). We then computed the M_{ext} as a percentage of M_{peak}. We found that, on average, the exo moment at the time of peak lumbar moment was 89 ± 4% of the peak exo moment (i.e., 11% lower than the peak exo moment generated by the elastic bands over the entire lifting cycle). We applied this relationship when analyzing the case series data, and thus assumed that M_{ext} was 89% of M_{peak} for each participant.

$$M_{exo} = M_{ext} - M_{flex} = .89(M_{peak}) - M_{flex} \qquad \text{Equation A2}$$

Fourth, we estimated M_{flex}, the lumbar flexion moment due to the trunk-worn weight of the elastic exosuit in this study. We found a high-end estimate by computing the flexion moment for a body

posture with 90 degrees of trunk flexion. We estimated the flexion moment from the exo weight (M_{flex}) by multiplying the trunk-worn weight of the SABER exosuit (0.62 kg) by the horizontal distance from the lumbar spine to the center of mass of this weight (40 cm for the case study participant). We found M_{flex} to be 2.5 Nm and used this estimate for all participants in the case series.

Exo moment at peak lumbar moment (M_{exo}) was calculated individually for each participant in case series 1 and 2 using Equation A2. This exo moment, an estimate of how much an exo reduces peak back loading, was then used as the input to Exo-LiFFT (Modeling Evaluation 1) to calculate cumulative damage.

The limitations of this approach outlined in the Appendix are as follows. Although M_{peak} was computed for each individual participant in case series 1 and 2, the 89% relationship between M_{peak} and M_{ext} (Equation A2) was derived from a single participant in a motion analysis lab. This was necessary because the case series studies were conducted in the field (at a U.S. Army base) where motion capture and force plate instrumentation were not available. Nevertheless, we believe the relationship we found was adequate for estimating net exo moments (M_{exo}, i.e., Exo-LiFFT inputs) in this study. In addition, the M_{flex} of 2.5 Nm was a high-end estimate and would be lower for individuals who lifted with less than 90 degrees of trunk flexion; however, these effects are small relative to the exo moments (M_{exo}) in this study (15-42 Nm, Table A2). Addressing the first objective about exo effects on endurance was completely independent of this relationship in Equation A2, and it is clear from the modeling evaluations (Fig. 3) that conclusions related to the secondary question did not require highly-accurate estimates of exo moment. Any reasonable or approximate estimate of exo moment would have yielded the same conclusions in this particular study. Nonetheless, there are other experiments where more accurate estimates of exo moment are expected to be important (e.g., when comparing between multiple exos or tasks). Thus, this Appendix provides a recommended methodology and initial benchmark data to build upon, in addition to providing a detailed description of the calculations we performed in this study. A final limitation is that this specific relationship (i.e., the 89% in Equation A2) cannot and should not be used for other (passive or powered) back exos. However, the thought process and methods for characterizing exo assistance and peak back offloading, as well as the general form of Equation A2, should be generalizable to other passive and powered back exos and could be applied to find device-specific estimates of net exo moment at the time of peak lumbar moment for other exos. To summarize simply, these methods detailed in Appendix B provide a way to estimate how much a back exo actually reduces peak loading on the user's low back, which is a useful input for ergonomic assessment tools.

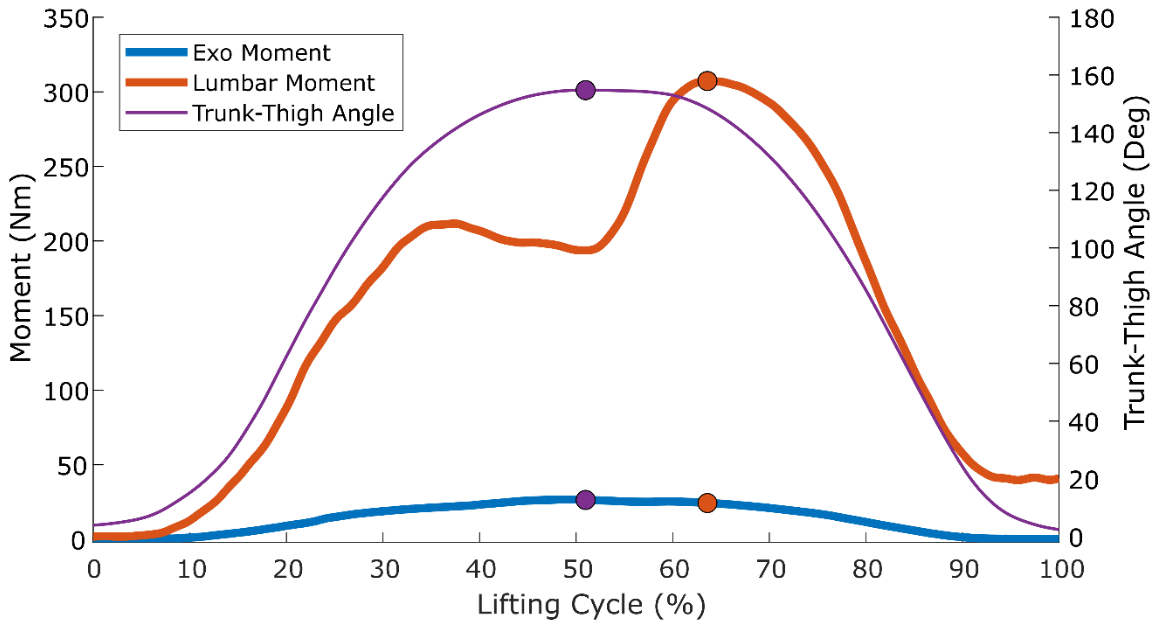

Figure A2. The lumbar moment (red), exo moment (blue), and trunk-to-thigh angle (purple) for a representative lifting cycle from the case study. The participant started in an upright standing posture (0-10% lifting cycle), bent down (10-50%), then lifted the object (50-90%) until they returned to standing posture while holding the object (90-100%). The peak exo moment (M_{peak}) occurs at the purple dots (near 50% lift cycle, the deepest part of the lift). The exo moment at peak lumbar moment (M_{ext}) occurs at the orange dots (near 65% lifting cycle). At the time of peak lumbar moment, the SABER exosuit assistance was about 89% of the magnitude of the peak exo moment.
